# MicroRNA-24-3p Targets Notch and Other Vascular Morphogens to Regulate Post-ischemic Microvascular Responses in Limb Muscles

**DOI:** 10.3390/ijms21051733

**Published:** 2020-03-03

**Authors:** Micol Marchetti, Marco Meloni, Maryam Anwar, Ayman Al-Haj-Zen, Graciela Sala-Newby, Sadie Slater, Kerrie Ford, Andrea Caporali, Costanza Emanueli

**Affiliations:** 1 Bristol Heart Institute, School of Clinical Sciences, University of Bristol, Bristol BS2 8HW, UK; micol.marchetti@gmail.com (M.M.); marcomeloni77@yahoo.it (M.M.); aymanzen@aol.com (A.A.-H.-Z.); G.Newby@bristol.ac.uk (G.S.-N.); sadie.slater@bristol.ac.uk (S.S.); kerrie.ford@gmail.com (K.F.); acaporal@exseed.ed.ac.uk (A.C.); 2 National Heart and Lung Institute, Imperial College London, London SW3 6LY, UK; maryam.anwar@lms.mrc.ac.uk; 3 BHF Centre for Cardiovascular Science, University of Edinburgh, Edinburgh EH16 4TJ, UK

**Keywords:** miR-24-3p, angiogenesis, endothelial cells, Notch, β-catenin, limb ischemia

## Abstract

MicroRNAs (miRs) regulate complex processes, including angiogenesis, by targeting multiple mRNAs. miR-24-3p-3p directly represses eNOS, GATA2, and PAK4 in endothelial cells (ECs), thus inhibiting angiogenesis during development and in the infarcted heart. miR-24-3p is widely expressed in cardiovascular cells, suggesting that it could additionally regulate angiogenesis by acting on vascular mural cells. Here, we have investigated: (1) new miR-24-3p targets; (2) the expression and the function of miR-24-3p in human vascular ECs; (3) the impact of miR-24-3p inhibition in the angiogenesis reparative response to limb ischemia in mice. Using bioinformatics target prediction platforms and 3′-UTR luciferase assays, we newly identified Notch1 and its Delta-like ligand 1 (Dll1) to be directly targeted by miR-24-3p. miR-24-3p was expressed in human ECs and pericytes cultured under normal conditions. Exposure to hypoxia increased miR-24-3p in ECs but not in pericytes. Transfection with a miR-24-3p precursor (pre-miR-24-3p) increased miR-24-3p expression in ECs, reducing the cell survival, proliferation, and angiogenic capacity. Opposite effects were caused by miR-24-3p inhibition. The anti-angiogenic action of miR-24-3p overexpression could be prevented by simultaneous adenovirus (*Ad*)-mediated delivery of constitutively active Notch intracellular domain (NICD) into cultured ECs. We next demonstrated that reduced Notch signalling contributes to the anti-angiogenic effect of miR-24-3p in vitro. In a mouse unilateral limb ischemia model, local miR-24-3p inhibition (by adenovirus-mediated miR-24-3p decoy delivery) restored endothelial Notch signalling and increased capillary density. However, the new vessels appeared disorganised and twisted, worsening post-ischemic blood perfusion recovery. To better understand the underpinning mechanisms, we widened the search for miR-24-3p target genes, identifying several contributors to vascular morphogenesis, such as several members of the Wingless (Wnt) signalling pathway, β-catenin signalling components, and VE-cadherin, which synergise to regulate angiogenesis, pericytes recruitment to neoformed capillaries, maturation, and stabilization of newly formed vessels. Among those, we next focussed on β-catenin to demonstrate that miR-24-3p inhibition reduces β-catenin expression in hypoxic ECs, which is accompanied by reduced adhesion of pericytes to ECs. In summary, miR-24-3p differentially targets several angiogenesis modulators and contributes to autonomous and non-autonomous EC crosstalk. In ischemic limbs, miR-24-3p inhibition increases the production of dysfunctional microvessels, impairing perfusion. Caution should be observed in therapeutic targeting of miR-24-3p.

## 1. Introduction

MicroRNAs (miRNAs, miRs) are small non-coding RNAs that regulate the expression of a wide number of target genes acting at posttranscriptional level. The canonical way of miR–targets interaction is via 3′-UTR binding, resulting in mRNA degradation, reduced RNA stability, or translational inhibition [1]. It is becoming apparent that miRs also bind to the amino acid coding sequence (CDS) and 5′UTR region of their targets. CDS binding has been associated with translational impairment and splicing regulation, while miR binding in the 5′UTR can even enhance the stability of the target [2].

Around 90 miRNAs have been identified to be hypoxia-responsive and involved in the post-ischemic angiogenesis responses [3,4,5]. We and others previously showed that miR-24-3p is susceptible to ischemia regulation and represses angiogenesis in the ischemic heart and developing zebrafish [6,7]. miR-24-3p is transcribed from two polycistronic units: miR-23a-27a-24-2 and 23b-27b-24-1. The miR-24-3p-1 and -2 transcripts produce identical mature miR-24-3p sequences. In contrast to miR-24-3p, both miR-23a/b and miR-27a/b reportedly promote angiogenesis [8]. The role of miR-24-3p in cardiovascular disease has caused some controversy, with Fiedler and us both reporting therapeutic advantage from miR-24-3p inhibition in the infarcted hearts [6,7], while Qian et al. described benefits from miR-24-3p overexpression [9]. Of relevance for the vascular system, miR-24-3p has been additionally shown to limit aortic vascular inflammation and to prevent murine abdominal aneurysm development [10]. However, the impact of endogenous miR-24-3p in the setting of limb ischemia has not yet been explored. When an ischemic insult occurs, the body reacts to restore oxygen and nutrients distribution [11,12,13]. Angiogenesis is a complex process, developing in different stages: pruning, sprouting, maturation, and stabilization. These different phases involve the cooperative work of vascular endothelial cells (ECs) and mural cells (pericytes and vascular smooth muscle cells—VSMCs), and the cell-to-cell coordination is mediated by secreted, membrane-bound, and intracellular signals such as the Notch and Wnt (wingless)/β-catenin signalling pathways [14,15]. In mammals, the Notch pathway involves four Notch receptors: Notch1, Notch2, Notch3, and Notch4 and five Delta-Serrate-Lag (DSL) ligands: Jagged-1, Jagged-2, Delta-Like 1(Dll-1), Delta-Like-3 (Dll-3), and Delta-Like-4 (Dll-4) [2]. The receptors and ligands are expressed on the extracellular membrane, and the Notch signalling is activated after the binding between receptors and ligands, which releases the Notch receptor intracellular domain (NICD) through sequential proteolytic cleavages by extracellular α-secretase (also known as ADAM17) and intracellular γ-secretase. Next, the NICD translocates into the nucleus and interacts with the transcriptional regulator CSL. This binding is essential to recruit transcription activators to the CSL complex and converts it from a transcriptional repressor into an activator, which then turns on several downstream effectors. Hairy and enhancer of split (HES) and HES-related repressor protein (HERP) genes encoding basic helix-loop-helix (bHLH) transcription regulators are target genes activated by this mechanism and are frequently used as reporters of Notch activity [16].

The Notch pathway is involved with several processes important for vascular morphogenesis; it regulates the individual responses and reciprocal communication of endothelial tip and stalk cell, which are essential for filopodia projection elongating the pre-existing vessels [17]. Moreover, Dll4 and Notch signalling couples sprouting angiogenesis and artery formation [17,18]. Additionally, Notch mediates vessel maturation through the ligand Jagged-1 that induces VSMCs’ differentiation and facilitates VSMC recruitment to endothelial cells (ECs), and the activation of the Notch pathway is engaged in response to ischemia, via hypoxia-inducible factor-α (HIF-α), VEGF and VEGF-receptor 2 [19,20,21]. There is a crosstalk between the Notch pathway and the Wnt/β-catenin signalling cascade. The Wnt signalling is involved in the regulation of angiogenesis-relevant functions such as cell determination, cell proliferation, and polarity acting directly on the stabilization of cell–cell interactions [16]. The Wnt family is represented by secreted glycoproteins able to activate either a canonical pathway, based on the Wnt and β-catenin interaction, or a non-canonical pathway, based on β-catenin-independent mechanisms [22]. The Wnt canonical signalling is based on the crucial role of β-catenin; when the signalling is not activated, β-catenin is expressed at the cytoplasmic level, where Dishevelled (Dvl, a family of protein which controls Wnt signalling pathways) inhibits the β-catenin activation. When the canonical pathway gets activated by binding between one of the Wnt ligands and the Frizzled receptor, the complex that keeps β-catenin in the cytosol gets phosphorylated and degraded, allowing β-catenin translocation into the nucleus, where it activates down-stream genes co-activating the T cell factor/lymphoid enhancer factor (TCF/LEF). Recently, a strong crosstalk between Wnt and Notch pathways has been observed in ECs, where NICD internalization was shown to induce the stabilization of β-catenin intra-nuclear activities [22,23,24]. The Wnt/β-catenin signalling is involved in the regulation of migration and adhesion mechanisms, as well in the cell–cell interactions mediating the stabilization of the adherens junctions [22,25]. Adherens junctions are cell–cell interactions’ regions that have a critical role in communication between cells and tissue organization and the regulation of the permeability of vascular structures. Adherens junctions are vascular endothelial (VE)-cadherin based complexes involving the catenin family represented by p120-catenin, β-catenin, and α-catenin. Once the signalling is activated by the binding between the extracellular domain of VE-cadherin with a distal membrane site composed of plakoglobin and α- and β- catenin, the catenin complex binds the actin filament inducing remodelling of the cytoskeleton [26,27]. VE-cadherin is involved in adult neo-vessels formation and vascular integrity maintenance [28].

Interestingly, miRs derived from the polycistronic miR-23a-27a-24 and 23b-27b-24 complexes are predicted to target the major endothelial junctional proteins. This led Li et al. to focus on miR-23a and miR-23b, demonstrating that miR-23a inhibits vascular permeability and miR-23b controls angiogenesis [29]. Li et al. did not expand their work to study miR-24-3p in EC junctions. However, Kourtidis et al. found miR-24-3p is involved in the regulation of epithelial junctions’ stabilization [30]. In detail, Kourtidis et al. showed that in the adherens junctions of epithelial cells, the cadherin complexes recruit the core components of the RNA-induced silencing complex (RISC), as well as a set of 522 messenger RNAs (mRNAs) and 28 mature miRNAs, including miR-24-3p, to regulate junction’s stabilization. Wnt/β-catenin was one of the top canonical pathways represented by these mRNAs. Moreover, Kourtidis et al. found MYC, which is known to be a downstream gene of the Wnt pathway, to be a direct target of miR-24-3p [30].

Here, we have identified that miR-24-3p targets the Notch pathways, regulating angiogenesis. Unexpectedly, while miR-24-3p inhibition improves vascularization in the ischemic limb muscles, this approach simultaneously compromises blood flow recovery.

## 2. Results 

### 2.1. miR-24-3p Targets Dll1/Notch Signalling and Regulates Vascular Cell Behaviour

**Figure 1:**
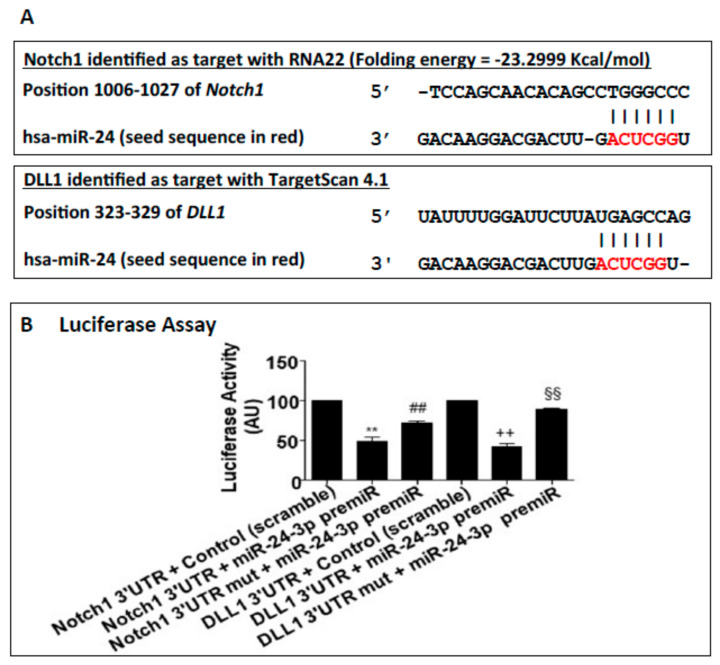
Target-gene prediction and primers sequences: (**A**) Analysis of the seed sequence of miR-24-3p (red letters) in the 3′-UTR of Notch1 and Dll1 with RNA22 software and Targetscan 4.1. Gray part: binding strength. (**B**) To validate the relation between miR-24-3p and the predicted targets, the luciferase assay was performed. HEK293 cells were transfected with pre-miR-24-3p or control and the reporter gene for Notch1 3′-UTR or Dll1 3′-UTR or their respective mutants. Experiments were performed in triplicate and repeated three times. Values are means ± SEM. ** *p* < 0.001 vs. Notch1 3′-UTR + control; ^# #^ *p* < 0.01 *vs.* Notch1 3′-UTR + pre-miR-24; ^++^ *p* < 0.05 vs. Dll1 3′-UTR+ control; ^$$^ p < 0.01 vs. Dll1 3′-UTR + pre-miR-24.

**Figure 2:**
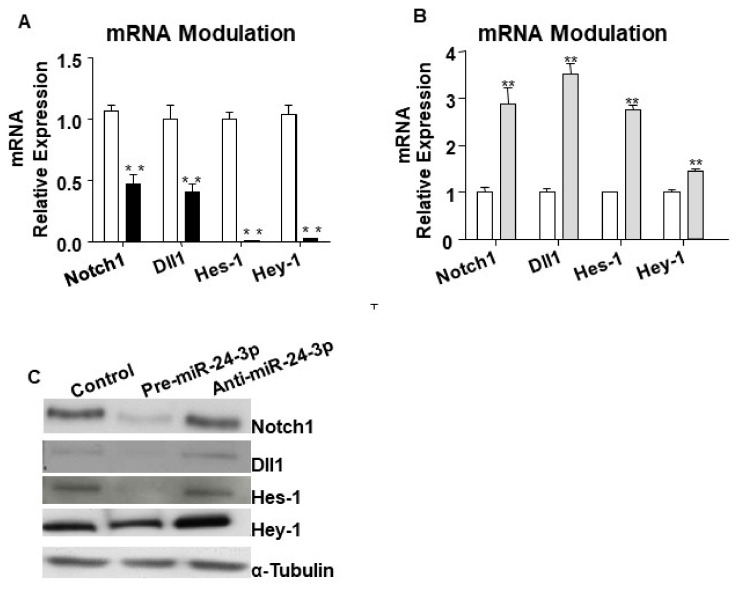
Notch1 and Dll1 direct targets of miR-24-3p and Notch pathway modulation in human umbilical vein endothelial cells (HUVECs). To validate the relation between miR-24-3p and the predicted targets Notch1 and Dll1, mRNA and proteins analyses were performed in HUVECs transfected with premiR-24-3p, anti-miR-24-3p, or control. Hes-1 and Hey-1 were also evaluated, as reporters of the Notch signalling activity. Bar graph (**A**) shows HUVEC transfected with pre-miR-24-3p (black columns) compared to control (scramble, white columns). Bar graph (**B**) shows HUVEC transfected with miR-24-3p inhibitor (light grey columns) compared to control (white columns). mRNA levels were normalised to 18S and quantified employing the 2^ΔΔ*C*t^ method and is presented as changes from the values in the respective control group. Experiments were performed in triplicate and repeated three times. Values are means ± SEM. Τ ** *p* < 0.01 vs. Notch1 3′-UTR + control. Panel (**C**) shows protein expressions of Notch1, Dll1, Hes-1, and Hey-1 forcing and inhibiting the expression of miR-24-3p. For Western blot analyses, α tubulin was used as house-keeping protein.

**Figure 3:**
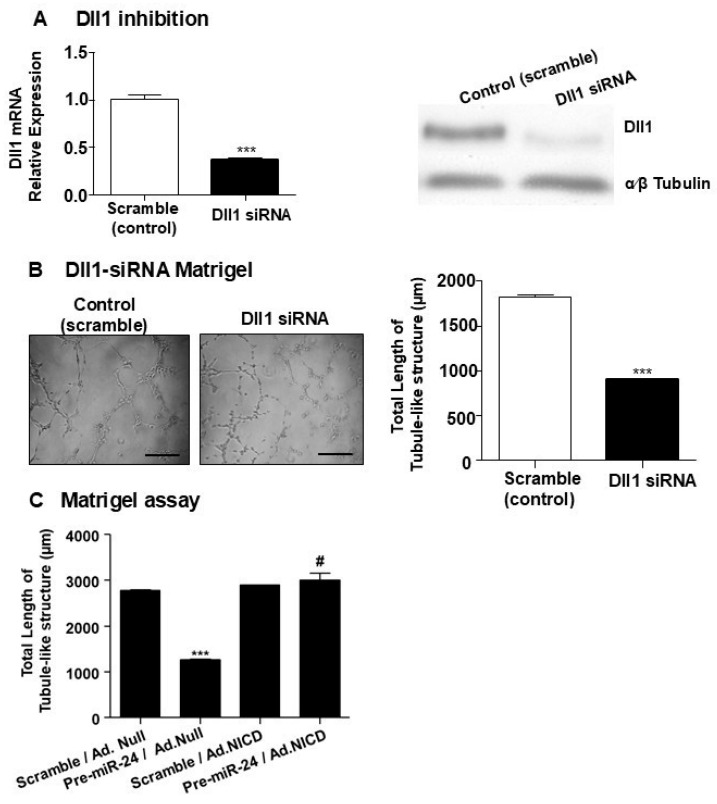
Dll1 inhibition in HUVECs affects the network-formation capability but NICD overexpression rescues it. Left bar graph (**A**, **left**) Dll1 mRNA relative expression in HUVECs transfected with Dll1-siRNA (black column) compared to HUVECs transfected with a scramble sequence (control). Dll1 mRNA expression was corrected to 18S and data were compared to the control group by the 2-ΔΔCt formula. Values are means ± SEM. *** *p* < 0.005 vs. scramble (**A**, **right**) Western blot bands of Dll1 protein and housekeeping α β-tubulin protein in HUVECs transfected with Dll1-siRNA compared to HUVECs transfected with a scramble sequence (control). (**B**, **left**) Photomicrographs of representative fields show the endothelial networks formed by HUVECs transfected with Dll1-siRNA compared to the control group (Scramble). (**B**, **right**) The bar graph represents the quantification of the total length of tube-like structures obtained in HUVECs transfected with Dll1-siRNA (black column) compared to the control group (scramble, white column). Values are means ± SEM. *** *p* < 0.005 vs. Scramble (**C**) The bar graph represents the quantification of the total length of tube-like structures in HUVECs treated as indicated: HUVECs transfected with scramble and infected with *Ad.Null*; transfected with pre-miR-24-3p and infected with *Ad.Null*; transfected with scramble and infected with *Ad.NICD* and HUVECs were transfected with pre-miR-24-3p and infected with *Ad.NICD*. Experiments were performed in triplicate and repeated 3 times. The raw data for the Panel (**C**) graph are presented in Supplemental Table S1. *** *p* < 0.005 vs. Scramble/*Ad.Null*; # *p* < 0.05 vs. miR-24 precursor/*Ad.Null*.

Bioinformatics analyses suggested Notch1 and the Notch ligand Dll1 to be potential targets of miR-24-3p (Figure 1A,B). Following the target genes prediction, Notch1 and Dll1 were confirmed as direct targets of miR-24-3p by using 3′-UTR gene report assays. As shown in Figure 1B the luciferase activity of the 3′-UTR of both Notch1 and Dll1 was significantly reduced by pre-miR-24-3p compared to the scramble controls. Moreover, luciferase activity was restored by mutations in the predicted 3′-UTR sites (for either Notch-1 or Dll-1) for miR-24-3p binding, highlighting the specificity of the assay. As expected, transfection of human umbilical vein endothelial cells (HUVECs) with pre-miR-24-3p increased the level of miR-24-3p (comparisons vs. scramble), and transfection with anti-miR-24-3p reduced miR-24-3p expression (Supplementary Figure S1A). We next validated the impact of forcing miR-24-3p expressional changes in Notch-1 and Dll-1 expression in HUVECs. HUVECs transfected with pre-miR-24-3p showed lowered mRNA (Figure 2A) and protein (Figure 2C) levels for Notch1 and Dll1. This was paralleled by inhibition of the Notch signalling, as revealed by reduced Hes1 and Hey1 expression in pre-miR-24-3p-treated HUVECs (vs. scramble control, Figure 2A,C). The opposite results were induced by use of anti-miR-24-3p in HUVECs (Figure 2B,C). We have already published that miR-24-3p affects microvascular EC biology [5]. These previous results are confirmed here in HUVECs. miR-24-3p transfection with pre-miR-24 miR-24-3p increased apoptosis (Supplementary Figure S2A), decreased proliferation (Supplementary Figure S2B), and reduced the in vitro angiogenesis capacity (assessed in Matrigel assays) (Supplementary Figure S2C) compared to scramble control. By contrast, miR-24-3p inhibition reduced apoptosis and increased proliferation compared to scramble controls (Supplementary Figure S2A,B,C). We next confirmed that silencing of Dll1 (validated at mRNA and protein level in Figure 3A) reduced the angiogenic potential of ECs (Figure 3B). Moreover, we obtained evidence that the antiangiogenic action of miR-24-3p could be prevented by rescuing Notch signalling via concomitant overexpression of a constitutively active Notch-1 intracellular domain (NICD) (Figure 3C). The recent data suggest that the antiangiogenic action of miR-24-3p could be mediated by the miR-24-3p-induced depression of the Notch signalling.

### 2.2. The Endothelial Expression of miR-24-3p and its Dll1 and Notch-1 Targets is Regulated by Hypoxia and Limb Ischemia

**Figure 4:**
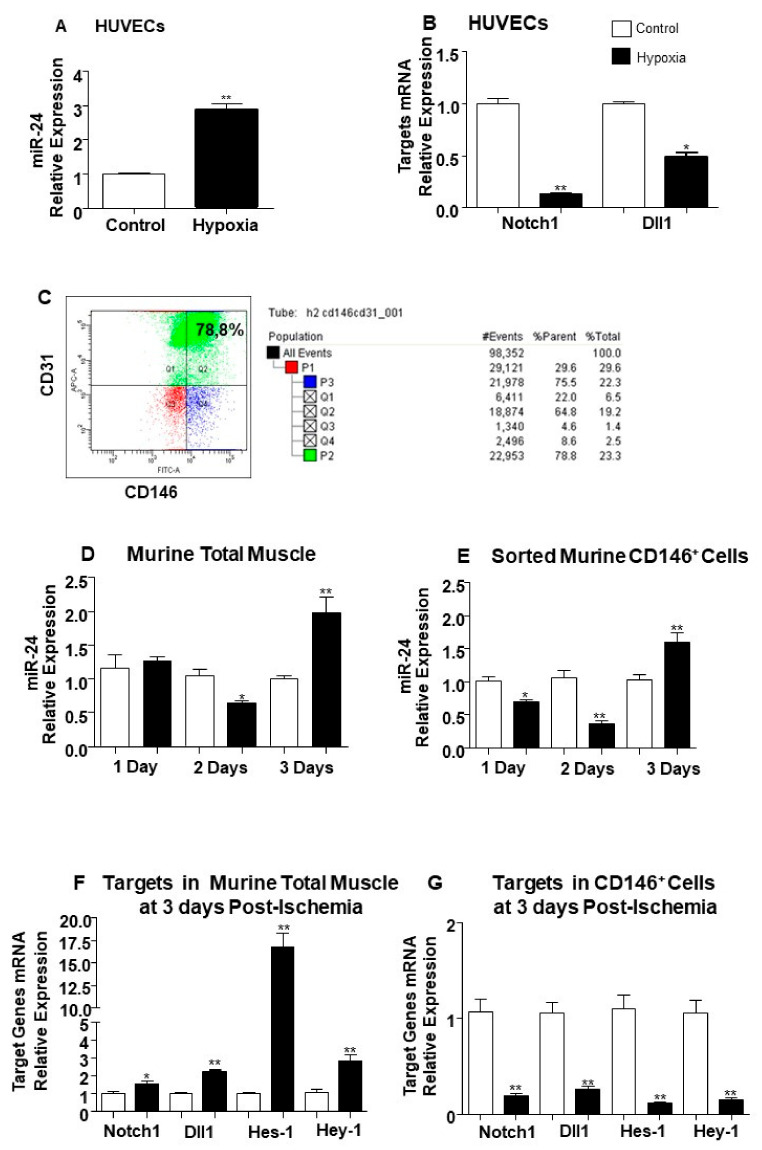
Modulation of miR-24-3p, Notch-1, and Dll1 mRNA expression by in vitro hypoxia and limb ischemia. (**A**) Bar graph shows miR-24-3p expression changes induced in endothelial cells (ECs) by culture for 48 h in hypoxia (1% O_2_, black column) versus standard cell culture conditions (21% O_2_, white column). (**B**) Bar graph shows Notch1 mRNA and Dll1 mRNA modulation by hypoxia in the same cells preparations used for the analyses shown in Panel A. (**C**) FACS analysis plot shows murine sorted CD146^+^ cells stained with FITC-conjugated CD146, APC-conjugated CD31 antibodies. The cells were extracted from pools of two adductor muscles. (**D**) Bar graph shows miR-24-3p expressional changes in murine total muscle samples at 1, 2, and 3 days post-ischemia (black columns) compared to murine non-ischemic control samples at matched time-point (white columns). (**E**) Bar graph shows miR-24-3p modulation in murine ECs sorted from total adductor muscle samples at 1, 2, and 3 days post-ischemia (black columns) compared to contra-lateral non-ischemic samples (white columns). (**F**) Bar graph shows miR expressional changes in Notch1, Dll1, Hes-1, and Hey-1 in mouse muscle harvested at 3 days post-ischemia (black column) compared to contra-lateral non-ischemic samples (white column). (**G**) Bar graph shows mRNA expression changes in Notch1, Dll1, Hes-1, and Hey-1 in murine ECs sorted from adductor muscle at 3 days post-ischemia (black column) compared to murine ECs sorted from non-ischemic muscles (white column). miR-24-3p expression was normalised to Snu6, and data are compared to the matched time-point group by the 2^ΔΔ*C*t^ method. Notch1, Dll1, Hes-1, and Hey-1 mRNA expression values were corrected to 18S. Experiments were performed in triplicate and repeated three times. Values are means ± SEM. *p < 0.05, **p < 0.01 vs. control matched time-point samples (*n* = 10 each group).

Next, we investigated whether the expression of miR-24-3p and its target genes Notch1 and Dll1 was regulated by hypoxia. Hypoxia (%1 O_2_) increased miR-24-3p levels in HUVECs compared to standard culture conditions (Figure 4A). Moreover, HUVECs cultured under hypoxic conditions showed a significant reduction of Notch1 and Dll1 (Figure 4B). In contrast to ECs, in pericytes, hypoxia did not affect miR-24-3p expression (Supplementary Figure S3).

Next, we assessed the regulation of miR-24-3p, Notch-1, and Dll-1 in muscle-resident microvascular cells exposed to hypoxia in the setting of mouse limb ischemia. CD31^+^ and CD146^+^ enriched ECs cells were isolated from mouse limb muscles after muscle digestion, as confirmed by flow cytometry (Figure 4C). Expressional changes in whole muscles were also analysed for reference. In the first 3 days from limb ischemia induction, miR-24-3p expression fluctuated in both total muscles (Figure 4D) and muscular ECs (Figure 4E). At 3 days post-surgery, miR-24-3p levels were higher in both the whole muscles and the ECs. Notch-1 and Dll-1 expression followed ischemia-induced miR-24-3p changes in the muscular microvascular cells, but not in the whole muscles (Figure 4F,G), suggesting that endogenous modulation of miR-24-3p impacts on the regulation of alternative molecular pathways in the non-vascular component of the muscle.

**Figure 5:**
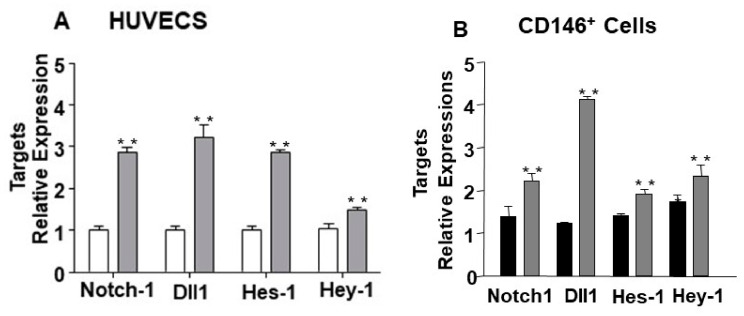
Impact of miR-24-3p inhibition on Notch pathway components’ miR-24-3p expression in cultured HUVECs and microvascular ECs exposed to limb ischemia. (**A**) Bar graph shows miR-24-3pNotch-1, Dll1, and down-stream targets Hes-1 and Hey-1 mRNAs in HUVEC transfected with miR-24-3p inhibitor (light grey columns) compared to control (white columns). (**B**) Bar graph shows the modulations of Notch-1, Dll1, Hes-1, and Hey-1 mRNA in CD146 positive cells isolated by ischemic adductor muscle at 3 days post-ischemia treated with in vivo miR-24-3p inhibitor (Ad.*Decoy.miR-24-3p,* grey bar) compared to scramble control (*Ad. Null*, black bar). Gene expression was normalised to 18S. Cell experiments were performed in triplicate and repeated three times. Analyses on muscular cells were developed using *n* = 10 mice per group. Values are means ± SEM. ** *p* < 0.005 vs. the respective control.

We next moved to validate the dependence of Notch-1 and Dll-1 expression by miR-24-3p in vascular cells cultured under hypoxia and in muscular ECs exposed to ischemia. miR-24-3p inhibition by anti-miR increased Notch-1 and Dll-1 in hypoxic HUVECs (Figure 5A). For the in vivo experiments, miR-24-3p action was inhibited by local delivery of *Ad. Decoy-miR-24-3p,* which increased Notch-1 and enhanced Notch signalling (Figure 5B).

### 2.3. miR-24-3p Inhibition Induces the Development of Dysfunctional Microvessels and Worsens Blood Flow Recovery in the Limb Ischemia Model

**Figure 6:**
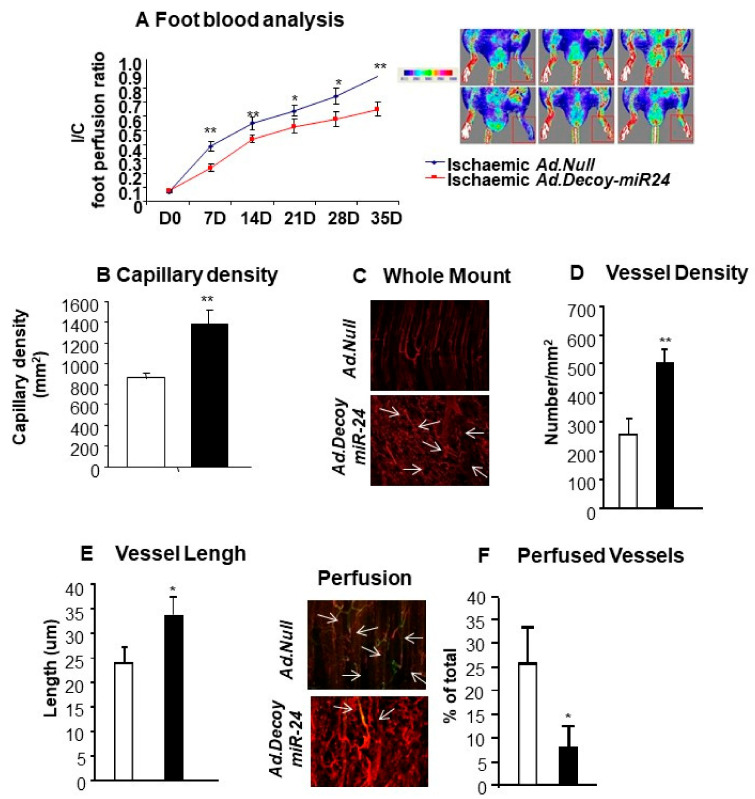
Effects of miR-24-3p inhibition in the ischemic limb. (**A**) Line graph shows the blood flow analysis in ischemic mice injected with *Ad.Null* (blue line) or *Ad.Decoy-miR-24-3p* (red line) (*n* = 12 animals per each group). (**B**) Bar graph shows increased capillary density after *Ad.Decoy*-mediated inhibition of miR-24-3p in the ischemic adductor muscle. Photomicrographs (**C**) and bar graphs (**D** and **E**) show the effect of miR-24-3p inhibition on the vascular organization (**C**), density (**D**), and length (**E**) in the ischemic adductor muscle. Photomicrographs and bar graph (**F**) show the percentage of perfused vessels in muscles treated with either *AdDecoy.miR-24-3p* or Ad.Null at 21 days of ischemia. *Ad.Decoy.miR-24-3p* treatment is shown in black, *Ad.Null* in white. Values are means ± SEM. ** *p* < 0.01 and * *p* < 0.01 vs. *Ad.Null*.

We next moved to test the therapeutic potential of miR-24-3p inhibition in the mice limb ischemia model. Unexpectedly, in comparison to the *Ad.Null* control, *Ad.Decoy-miR-24-3p* consistently impaired blood perfusion recovery overtime (Figure 6A). According to microscopic analyses at 3 weeks post-surgery, in the *Ad.Decoy-miR-24-3p* group, the reduced blood perfusion was associated with increased capillary density in the ischemic muscle (Figure 6B), increased vessel density (Figure 6C,D), and increased vessel length (Figure 6E). However, the number of perfused vessels was reduced by miR-24-3p inhibition (Figure 6F).

### 2.4. miR-24-3p Targets the Expression of β-Catenin and Other Vascular Morphogens

**Figure 7:**
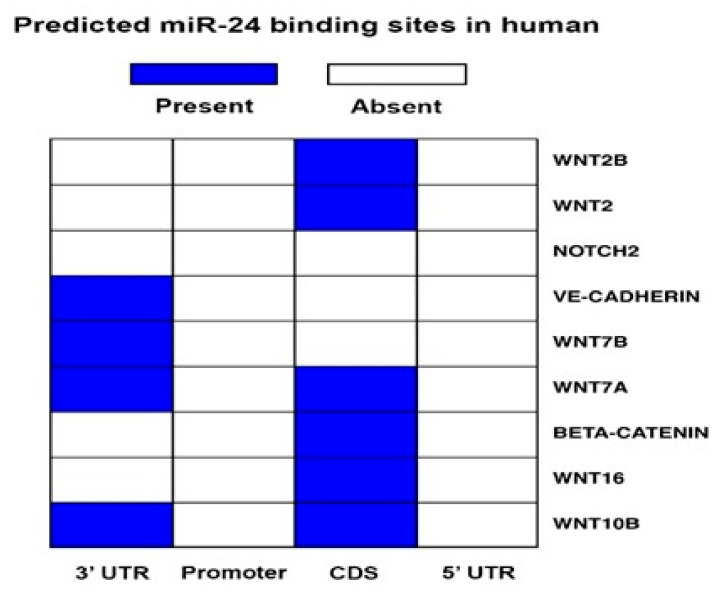
miR-24-3p and Wnt/β-catenin binding prediction. The table shows the target genes prediction related to the Wnt/β-catenin pathway in human (left) and mouse. The prediction was performed using mirWalk software.

**Figure 8:**
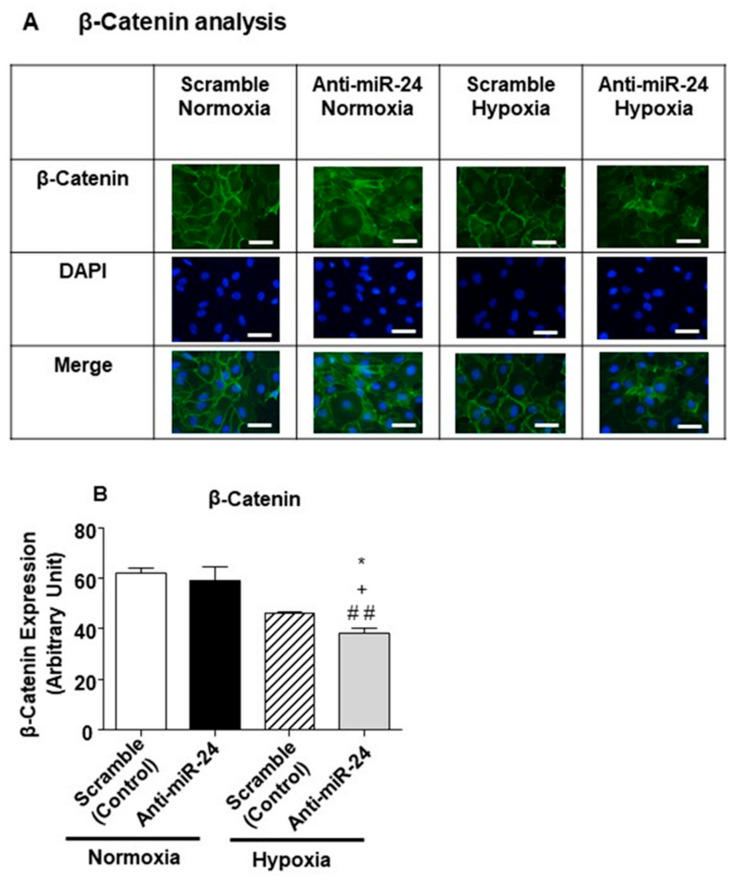
β-catenin modulation in in vitro model of hypoxia. (**A**) The panel shows representative pictures of β-catenin staining in HUVECs treated with miR-24-3p inhibitor and exposed to hypoxia (1% O_2_) or kept under standard culture conditions for 48 h. Scale bars corresponds to 50 μm (**B**) Bar graph shows the quantification of the staining, where the black bar represents HUVECs treated with miR-24-3p inhibitor, and the white bar represents HUVECs treated with scramble (transfection control). Adhesion assay was performed in quintuplicate and repeated two times; β-catenin staining was performed in triplicate and repeated three times. Values are means ± SEM. * *p* < 0,05 vs. scramble hypoxia, ^+^ *p* < 0.05 vs. anti-miR-24-3p in standard culture conditions; ^##^ *p* < 0.01 vs. scramble in standard culture conditions.

To investigate the mechanism behind the development of aberrant vessels after miR-24-3p inhibition in limb muscles, we next evaluated the possibility that miR-24-3p inhibits the expression of additional morphogens. Figure 7 shows that Notch2, several Wnts, Ve-cadherin, and β-catenin could also all be targeted either in 3′-UTR or in the coding sequence regions (depending on the genes). We then decided to investigate the impact of miR-24-3p on β-catenin [31,32]. It was suggested that while sites located in the 3′-UTR sites are effective in down-regulating the mRNA levels, miRNA binding sites that are located in the CDS are effective in inducing translational inhibition [33,34]. Therefore, we analysed the impact of miR-24-3p inhibition at the protein level by performing immunocytochemical analyses. However, contrary to expectations, under hypoxic conditions, miR-24-3p inhibition reduced β-catenin protein and mRNA expression in ECs (Figure 8A,B). This response, which was possible due to an indirect action of miR-24-3p on β-catenin production of degradation, could partly justify the formation of anatomical and functional abnormal microvessels in response to miR-24-3p inhibition under ischemic conditions.

We finally identified that miR-24-3p inhibition could also disturb VEGF-R2 expression in hypoxic ECs (Supplementary Figure S4A,B). We observed VEGF-R2 expression to be reduced in total muscle and CD146^+^ cells at 3 days post-limb ischemia induction; however, the in vivo treatment with *Ad.Decoy.miR-24-3p* miR-24-3p did not rescue the expression compared to the non-ischemic control (Supplementary Figure S4C).

Supplementary Figure S5 presents the proposed model of the impact of miR-24-3p on the interaction between Notch and β-catenin in hypoxic endothelial cells.

### 2.5. miR-24-3p Inhibition in ECs Impairs Their Capacity to Adhere with Pericytes

**Figure 9:**
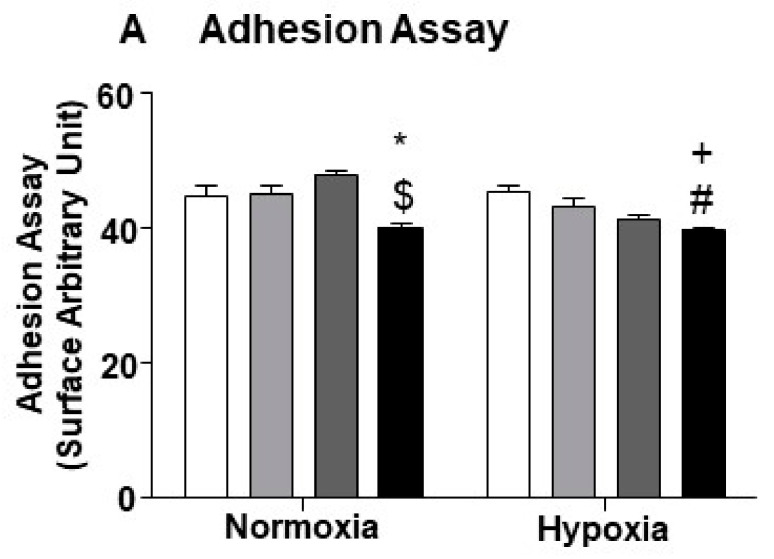
Reduced miR24 in ECs affects the adhesion between ECs and pericyte. miR-24-3 under standard (5% O_2_) or hypoxic culture conditions. Groups are pre-miR-24-3p transfected ECs miR-24-3p (black column); scramble control (dark grey column); vehicle control of transfection (light grey); and untreated cells (white column). Adhesion assay was performed using five wells per treatment and repeated two times. Values are means ± SEM. * *p* < 0.05 vs. untreated cells in normoxia; + *p* < 0.05 vs. untreated Cells in hypoxia; $ *p* < 0.05 vs. scramble in normoxia; # *p* < 0,05 vs. scramble control in hypoxia.

Pericytes wrap blood microvessels, playing important roles in vascular morphogenesis, maturation, and stability [32]. After having observed that hypoxia does not alter miR-24-3p expression in pericytes (Supplementary Figure S3), we hypothesised that altered mR-24 level in ECs could affect the adhesion between the two vascular cell types, thus compromising the formation of robust microvessels. In line with this hypothesis, we found that miR-24-3p inhibition impairs the ECs interaction with mural cells. In fact, when prompted in cell-to-cell adhesion assays, miR-24-3p deprived ECs showed reduced capacity to adhere with co-cultured pericytes (Figure 9).

## 3. Discussion

miR-24-3p expression has already been reported by us and others to be increased in ischemic tissues and cells [5,6,7] and to be regulated by HIF-1α [6,34,35,36,37,38]. This study has investigated the role of miR-24-3p in post-ischemic peripheral revascularization. Peripheral ischemia affects almost 12% of the adult Western world population and represents a severe clinical condition, characterised by the lack of oxygen and nutrients in the lower limb, conditions that over time lead to the amputation of the limb. Using an in vivo model of limb ischemia, we have characterised the local inhibition of endothelial miR-24-3p function and the consequential activation of the early phases of angiogenesis as confirmed by increased vascular density assessed through histological analysis. However, both vessel maturation and stabilization were impaired. With our study, we consolidated the knowledge related to miR-24-3p as hypoxia-regulated miR [27,28,29,39,40], highlighting its involvement in the modulation of gene expression in human ECs cultured under hypoxic conditions as well as in ECs harvested from a murine hind-limb ischemia model. Previous studies already demonstrated the negative impact of miR-24-3p in cellular processes associated with angiogenesis [5,6]. We have identified and validated that miR-24-3p binds to and regulates expression of two important players of the Notch signalling: Notch-1 and Dll1. The Notch pathway is known to be involved at the early stage of the complex angiogenic mechanism, when from the pre-existent structures, following pruning, new vessels start to take shape in a finely tuned mechanism involving the coordination between the stalk and tip cells. We assessed a significant increase of miR-24-3p in ECs exposed to hypoxia. Increased miR-24-3p was associated with decreased expression of Notch1 receptor and Dll1 ligand and impaired Notch signalling under hypoxic and ischemic conditions. We were able to rescue Notch signalling in hypoxic ECs by inhibiting miR-24-3p, in vitro and in vivo. This approach produced an increased vascularization of the murine ischemic limb muscles. However, the increased neo-genesis was unexpectedly associated with reduction of intra-vascular blood flow and tissue perfusion. The three-dimensional analysis of the tissue showed twisted, highly convoluted, and poorly perfused neo-structures highlighting the lack of organization and maturation of the new structures. Notch is known as one of the most important pathways controlling cell-fate determination angiogenesis and arteriogenesis.

In this previous study, the “Notch effect” was mediated by response to nitric oxide-angiopoietin signalling [41]. Our earlier results suggest that Dll4 activation early on in revascularization can lead to unproductive angiogenesis and arteriogenesis, despite increased vascular densities. We hypothesised that a spatial and temporal balance of growth factors needs to be perfected for ideal functional and anatomical revascularisation [42]. Notch is one of a few important and interacting pathways regulating vascular morphogenesis. Tissue repair requires a tightly regulated spatiotemporal control of gene expression, and several highly conserved signalling pathways have evolved to mediate such communication events across different bodily tissues. Among these, the Notch, Wnt-catenin, and hypoxia signalling pathways, which are relevant to our data, play critical roles (reviewed in [43,44,45]). When these pathways are unbalanced, this can induce disease and disrupt endogenous repair mechanisms [45], as can be seen in this paper in relation to post-ischaemic angiogenesis. Notch receptor–ligand interaction results in cleavage events catalysed by extracellular ADAM metalloproteases and an intracellular γ-secretase-containing complex releasing the NICD. Once released, the NICD translocates into the nucleus where it forms a complex able to regulate the expression of target genes. Therefore, we decided to investigate the possibility that miR-24-3p acts on additional morphogens. Interestingly, the bioinformatically predicted targets of miR-24-3p included members of the Wnts, Ve-cadherin and β-catenin. The Wnt/β-catenin cell-signalling is involved in the stabilization and maturation of the new structures through adherens junction formation [17,18,19,20]. We found a decreased expression of β-catenin and VEGF-R2 in hypoxic HUVECs treated with anti-miR-24-3p. Moreover, a previous study in rats identified the regulation of Wnt4 signalling pathway in a rat carotid injury model, where the therapeutic overexpression of miR-24-3p could reduce vascular smooth muscle cells’ (VSMCs) proliferation and neointima formation by inhibiting Wnt4, Dvl-1 (which belongs to the Dvl family of proteins involved in Wnt and β-catenin signalling pathways), and β-catenin [46]. This in turn led to regulation of the expression of Cyclin D1 and p21 [44] and hence VSMC proliferation by miR-24-3p. In contrast to hypoxic ECs, VSMCs from injured carotids expressed a lower endogenous level of miR-24-3p. The regulation by endogenous miR-24-3p of vascular morphogenesis under hypoxia was also confirmed by Kasper et al. [46]. Working with zebrafish mutants, this group showed that vertebrate miRNAs provide tissue robustness to changing environments in development and that loss of miR-24-3p family increases the variance of developing vascular traits [46]. The miR-24-3p fish mutants appeared broadly sensitised to vascular stresses, including hypoxia, and to angiogenesis acting drugs. In line with our results, these drugs were targeting the Wnt and Notch signalling (BIO: pro-angiogenic drugs Wnt signalling agonist; GSI: proangiogenic Notch signalling antagonist; SU5416: anti-angiogenic VEGF-R2 antagonist) [46]. Interestingly, The Notch and Wnt signal transduction pathways can regulate each other. As an example, the Wnt pathway reportedly regulates *Dll1* expression. Moreover, the Notch target gene *Hes1* is also regulated by β-catenin-mediated Wnt signalling [47]. Furthermore, the direct interaction between β-catenin and Notch-1 reduces the ubiquitination of Notch-1, which results in increased expression of *Hes1* [48]. Moreover, NICD interacts with HIF-1α. [49] In line with our functional results, the Notch/β-catenin interaction was reported to support angiogenesis via its synergic occurrence at several target genes [44]. However, additional models of Notch/β-catenin interactions are present in literature, and endogenous Notch-1 has been reported to both negatively regulate β-catenin stability [45] and decrease the transcriptional activity of a β-catenin-responsive reporter construct, suggesting that Notch could dampen β-catenin-mediated responses to Wnt. Our results indicate that the model of interaction between Notch and Wnt/β-catenin is further complicated by the interference of microRNAs. Here, we have provided evidence that miR-24-3p is regulated by hypoxia, which in turn modulates both the Notch and Wnt-catenin pathways affecting the expression of individual components of both systems. This is particularly important when considering that there is considerable evidence for crosstalk between Notch and hypoxia signalling. In fact, the physical interaction of NICD-1 and nuclear HIF1α upregulates a subset of target genes for Notch [50]. Moreover, cytoplasmic HIF-1α increases the activity of the γ-secretase complex [46], increasing the release of NICD and the expression of the target gene *Hes1.* The crosstalk between Notch signalling and the hypoxia response has been shown to be critical in balancing sprouting angiogenesis and vascular remodelling [46,47,48,49,51,52,53,54]. In this respect, Notch signalling regulates the expression and/or activities of the VEGF receptors and ligands and it might expand to control pericyte recruitment during blood vessel formation by affecting the PDGF-B-PDGFRβ dynamics [49,51,53,55]. In line with all the above and with the results obtained by this study, we already provided recent evidence that an unbalanced enhancement in Notch signalling results in unproductive revascularization in a mouse model of peripheral ischemia [37].

These data, taken together, highlight the complexity of the whole angiogenic process under miR-24-3p control. This should be a caution for the therapeutic use of miR-24-3p inhibitors in peripheral ischaemia and possibly under different pathogenic conditions. In fact, miR-24-3p emerges as an important microRNA in different pathological scenarios with an ischemic component, such as heart, lung, pancreas, and brain diseases [5,6,7,34].

## 4. Material and Methods

### 4.1. Cell Culture

Human umbilical vein endothelial cells (HUVECs) were purchased from Lonza and routinely grown in EGM-2 plus 2% FBS. SMCs (Lonza, Basel Switzerland), were cultured in DMEM with 10% FBS. Human pericytes were prepared from the saphenous vein of six human donors, as previously extensively described [56]. For experiments under hypoxic conditions, cells were cultured in normal growing medium and exposed to 1% O_2_ for 72 h and compared to control cells kept in standard culture conditions (21% O_2_).

### 4.2. miR-24-3p Target Prediction and Target Genes Validation

To identify new direct target gene candidates of miR-24-3p (indicated as miR-24-3p for the rest of the manuscript), we employed several miR target prediction algorithms (TargetScan 4.1, miRanda, miRbase, Diana, PicTar, rna22, and mirWalk). In order to investigate whether miR-24-3p directly regulates the in silico predicted target Notch1 and Dll1 expression by 3′-UTR binding, portions of the 3′-UTR of these potential target genes were inserted downstream of a luciferase open reading frame. Notch1 3′-UTR and Dll1 3′-UTR vectors were purchased from Addgene (Cambridge, MA, USA). For controls, we prepared similar vectors in which five nucleotides mutations were inserted in the 3′-UTR sequences (mutations). The different luciferase constructs were transfected into HEK-293 cells together with either pre-miR-24-3p (AM17100, Ambion, MA, USA), or a negative control oligonucleotide sequence (scramble control). HEK-293 cells were chosen for their high efficiency of transfection and the absence of endogenous miR-24-3p expression. Cells were cultured for 48 h and assayed with the Dual-Luciferase Reporter assay System (Promega, WI, USA). In a subsequent stage of the project, for identification of additional target genes of miR-24-3p, we expanded our search to the CDS and/or 5′UTR regions’ binding sites.

### 4.3. RNA Extraction and TaqMan Quantitative Real-Time PCR Analysis

Total RNA was extracted from either cultured cells or tissues (vide infra) using the miRNeasy mini kit (Qiagen, Crawley, UK), and RNA concentration was quantified with the NanoDrop spectrophotometer. Quantitative real-time polymerase chain reaction (RT-qPCR) to measure relative miR expression was performed using the TaqMan miRNA reverse transcription kit and miRNA Assay (Applied Biosystems, Life Technologies, CA, USA). miR-24-3p expression was normalised to the U6 small nucleolar RNA (snRU6) (Applied Biosystem, CA, USA). For mRNA analysis, single-strand complementary DNA was synthesised from 10 ng of total RNA. Complementary DNA was amplified by real-time PCR and normalised to 18S ribosomal RNA (endogenous control) (Qiagen Reverse Transcription Kit, Crawley, UK). Each reaction was performed in triplicate. Quantification was performed by the 2^ΔΔ*C*t^ method [39]. Quantitative reverse transcription PCR was used to measure 18S ribosomal RNA, Notch1, Dll1, Hes-1, and Hey-1 (reported in Table 1) in HUVECs, pericytes, VSMCs, C2C12 cells, and ECs sorted form either the ischemic adductors or non-ischemic contra-lateral muscles.

### 4.4. Western Blot Analyses

Western blots for Notch1 (ab8925, dilution 1:500) and Dll1 (ab10554, dilution 1:500) from Abcam (Cambridge, UK); Hes-1 (GTX62458, dilution 1:1000) and Hey-1 (GTX82287, dilution 1:1000) from GeneTex (California, USA); and α-Tubulin (T9026, dilution 1:10000) from Sigma (Dorset, UK) were performed on HUVECs.

### 4.5. miR-24-3p Mimic and Anti-miR-24-3p Transfection

pre-miR-24-3p precursor (50 nM final concentration, pre-miR-24-3p, AM17100, Ambion, MA, USA), miR-24-3p inhibitor oligonucleotides (50 nM final concentration, Anti-miR-24-3p, AM17000, Ambion, MA, USA), and scrambled oligonucleotides (50 nM, final concentration, Cy™3 dye-labelled Anti-miR™ Negative Control, AM17011, Ambion, MA, USA) were transfected into HUVECs, VSMCs, and C2C12 using Lipofectamine 2000 Reagent (Invitrogen, Carlsbad, CA, USA) following the manufacturer instructions.

### 4.6. Dll1 siRNA Transfection

Dll1 siRNAs (50 nM final concentration, HS-Dll1, Qiagen) or scrambled oligonucleotides (50 nM final concentration, Ambion, MA, USA) were transfected into HUVECs using Lipofectamine 2000 Reagent (Invitrogen, CA, USA) following the manufacturer instructions.

### 4.7. Adenoviral Vectors

The adenoviral vector (Ad) carrying a decoy for miR-24-3p (*Ad.Decoy-miR-24-3p*) employed for this study has been discussed in our previously published work [6]. *Ad.Null* was used as control. Moreover, an Ad enabling expression of the Notch intracellular domain (*Ad. NICD*) was also used.

### 4.8. HUVEC Infections with Adenoviruses 

HUVECs were seeded (2 × 10^5 cells) in a six-wells plate and then infected with *Ad.Decoy-miR-24-3p*, *Ad. NICD* or *Ad. Null* (control) at 250 multiplicity of infection (MOI).

### 4.9. Biology Assays with HUVECs

Cell proliferation was measured by BrdU Incorporation assay (Promega, WI, USA) and apoptosis by Caspase-ApoGlo 3/7 assay (Promega, WI, USA). The impact of miR-24-3p on capillary-like cord formation was prompted in vitro using a Matrigel assay. HUVECs were transfected with pre-miR-24-3p or anti-miR-24-3p and control or infected with *Ad. Decoy-miR-24-3p* or *Ad. Null* (control). Next, cells were seeded in 96-well plates previously coated with growth factors-enriched Matrigel (Matrigel Matrix, Basement Membrane, BD, Oxford, UK). Endothelial network formation was quantified by morphometric analysis. Photomicrographs (40X) were taken 12 h after the start of the experiment: three photomicrographs were taken for each treatment, and each treatment was performed in triplicate. ImageJ was used to quantify the total length of the capillary-like structures. For cell migration assay, HUVECs were transfected with either anti-miR-24-3p or scramble control and seeded in a 12-wells plate to reach confluence by 48h. Then, in order to inhibit cell proliferation, cells were incubated with 2mM of hydroxyurea, and the monolayer was scratched. Images were taken at the beginning of the experiment (time zero, T0), 5, and 12 h. Five photomicrographs were taken for each treatment, and each treatment was performed in triplicate.

### 4.10. Pericyte Adhesion to HUVECs

HUVECs were transfected according to the previous method’s description and in vivo stained, according to the company protocol, with CellTracker™ Green CMFDA Dye (Lie Technologies, Carlsbad, CA, USA and then seeded overnight in a 96-wells plate at the density of 5000 cells/well. The experiment was simultaneously performed in either standard culture conditions (21% O_2_) or hypoxia (1% O_2_). The following day, pericytes were stained according to the company protocol with CellTracker™ CM-DiI Dye (Life Technologies) and left for 2 h in full media. The HUVEC-containing wells were then gently washed with medium before seeding of 5000 pericytes on the HUVECs layer. Photomicrographs (40X) were taken at 12h from pericyte seeding. For each treatment, five photomicrographs were taken, and each treatment was performed in triplicate. ImageJ was used to quantify the adhesion.

### 4.11. β-catenin Quantification

Immunofluorescent staining for β-catenin was performed on HUVECs transfected with either anti-miR-24-3p or scramble control. The cells were either exposed to hypoxia or kept under standard conditions for 72 h and then fixed with 4% PFA. Following incubation with blocking buffer, the samples were incubated with primary antibody (1:200, ab32572, Abcam) overnight at 4 °C. Further incubation with appropriate secondary antibody (1:400) was performed. Nuclei were stained with DAPI. Photomicrographs (40×) were taken, and ImageJ was used to quantify the β-catenin expression.

### 4.12. In Vivo Studies

The experiments involving mice were performed in accordance with the Guide for the Care and Use of Laboratory Animals issued by the US National Institutes of Health (No. 85-23, 2011) and comply with the ARRIVE guidelines and were carried out in accordance with the UK Animals (Scientific Procedures) Act 1986 and associated guidelines, EU Directive 2010/63/EU for animal experiments, and the National Institutes of Health guide for the care and use of Laboratory animals (NIH Publications No. 8023, revised 1978). Experiments were approved by the UK Home Office (PPL number 30/2811, approved on the 02/08/2011) to be held at the University of Bristol Ethics Committee 

### 4.13. Mouse Model of Unilateral Limb Ischemia and in Vivo Gene Transfer

To model limb ischemia in mice, 8-week-old male CD1 mice (Harlan) were anaesthetised (tribromoethanol, 880 mmol kg−1i.p., Sigma) to undergo left femoral artery ligation, followed by electrocoagulation, as previously described [7,57]. Three protocols were designed to respectively assess 1) the expressional changes in endogenous miR-24-3p and its target genes in the response to ischemia (control: non-ischemic muscles); 2) target gene changes induced by miR-24-3p inhibition via *Ad. Decoy-miR-24-3p* (10^9^ p.f.u.) in the ischemic limb (control group: *Ad. Null*); and 3) the impact of miR-24-3p inhibition by *Ad. Decoy-miR-24-3p* on post-ischemic angiogenesis and blood-flow (BF) recovery (controls: *Ad.Null*). For protocols 1 and 2, mice were sacrificed at 1, 2, and 3 days post-surgery and their ischemic and contra-lateral adductor limb muscles harvested for subsequent biochemical analyses on whole muscles or in ECs isolated from the muscles (see below). For protocol 3, either *Ad. Decoy-miR-24-3p* or *Ad. Null* (control) (10^9^ p.f.u.) was injected in three sites of the left adductor muscles [31]. The hindlimb blood-flow (BF) was measured immediately after surgery by colour laser Doppler (Moor) to confirm successful surgical induction of limb ischemia and as a baseline to calculate the recovery to the ischemic foot at 7, 14, 21, 28, and 35 days after ischemia. For histology, subgroups of mice were sacrificed at 21 days post-limb ischemia induction.

### 4.14. Expressional Analyses on Murine Tissues and Cells

Expressional studies were performed on the whole adductor muscle and microvascular ECs sorted from the muscle as previously published [7]. Briefly, the adductors from two mice were pooled to produce a total of *n* = 6 samples per group. Muscles were enzymatically digested, and the ECs were immunomagnetically sorted using a CD146 antibody (Beads CD146 mouse for cell sorting (LSEC, MACS, Surrey, UK). To verify EC enrichment, the single-cell suspension was incubated with FITC-conjugated CD146 and APC-conjugated CD31 antibodies or the respective isotype for negative control. Unstained and single stained controls were performed to define the positivity. The population of sorted ECs was circa 79% of the whole cell population sorted (as exemplification, see Figure 4C).

### 4.15. Histology and Immunohistochemistry

At 21 days after surgery and gene transfer, mice were perfusion-fixed, and adductor muscles were harvested for histological evaluation. OCT- and paraffin-embedded samples were prepared for histological analysis of capillary and arteriole density at 35 days post-ischemia. Capillary density was determined on muscular transverse sections using fluorescent microscopy on sections stained with Alexa 568-conjugated Isolectin-B4 (an endothelial cell marker), α-Smooth Muscle Actin (α- SMA, a vascular-cells marker), and DAPI (to identify the nuclei). Additional adductor muscles were OCT embedded and used for whole-mount immunohistochemistry analyses. In brief, adductor muscle was cut longitudinally into 100 μm-thick slices. Following incubation with blocking buffer, the samples were incubated with primary antibody overnight at 4 °C. Further incubation with an appropriate secondary antibody was performed. Serial z-stack images of adductor muscles were generated using confocal microscopy. At 21 days post-ischemia, whole mounts of muscles were prepared to further characterise vascular morphology. Investigation using whole mounts of mice which had been perfused with green-fluorescent B4-isolectin under physiological conditions 20 min previous to their sacrifice was carried out. Endothelial structures were marked by red fluorescent Isolectin-B4, and the whole mounts were then imaged using confocal microscopy and quantified using dedicated software (Volocity Software—PerkinElmer).

### 4.16. Statistical Analyses

Group differences of continuous variables were compared by one-way ANOVA or Student’s t-test as appropriate. Relationships between variables were determined by the Pearson correlation coefficient. Continuous data are expressed as mean ± SEM. A *p* value < 0.05 was considered statistically significant. Analyses were performed with GraphPad Prism 5.0.

## Figures and Tables

**Table 1:** Primers employed for mRNA analysis.

Gene	Murine Primers
18S	F 5′-TAGAGGGACAAGTGGCGTTC-3′ R 5′-TGTACAAAGGGCAGGGACTT-3′
Murine Notch1	F 5′-CCTTGCTCTGCCTAACGC-3′ R 5′-GGAGTCCTGGATCGTTGG-3′
Murine Dll1	F 5′-GCAGGACCTTCTTTCGCGTAT-3′ R 5′-AAGGGGAATCGGATGGGGTT-3′
Murine Hes1	F 5′-TCAACACGACACCGGACAAAC-3′ R 5′-ATGCCGGGAGCTATCTTTTCTT-3′
Murine Hey1	F 5′-CCGACGAGACCGAATCAATAAC-3′ R 5′-TCAGGTGATCCACAGTCATCTG-3′

## Supplementary Materials

The following are available online at https://www.mdpi.com/1422-0067/21/5/1733/s1
