# Correction: Marchetti et al. MicroRNA-24-3p Targets Notch and Other Vascular Morphogens to Regulate Post-ischemic Microvascular Responses in Limb Muscles. *Int. J. Mol. Sci*. 2020, *21*, 1733

**DOI:** 10.3390/ijms27115014

**Published:** 2026-06-02

**Authors:** Micol Marchetti, Marco Meloni, Maryam Anwar, Ayman Al-Haj-Zen, Graciela Sala-Newby, Sadie Slater, Kerrie Ford, Andrea Caporali, Costanza Emanueli

**Affiliations:** 1 Bristol Heart Institute, School of Clinical Sciences, University of Bristol, Bristol BS2 8HW, UK; micol.marchetti@gmail.com (M.M.); sadie.slater@bristol.ac.uk (S.S.);; 2 National Heart and Lung Institute, Imperial College London, London SW3 6LY, UK; 3 BHF Centre for Cardiovascular Science, University of Edinburgh, Edinburgh EH16 4TJ, UK

In the original publication [1], there were mistakes in Figure 3B,C, and Supplementary Figure S2C as published. For Figure 3B,C, the y-axes were wrongly labelled as “Dll1 mRNA relative expression”. In Figure 3C, the Matrigel panel was found to contain a duplicate image from Supplementary Figure S2C; this panel has been removed and replaced by Supplemental Table S1 (containing the original densitometry data), while Supplementary Figure S2C has been updated with a new Matrigel panel. Consequently, the legends for these figures have been corrected to properly reflect the experimental groups. The corrected Figure 3B,C, and Supplementary Figure S2C (including their legends) appear below. The authors apologise for the confusion and state that the scientific conclusions are unaffected. This correction was approved by the Academic Editor. The original publication has also been updated.

**Figure 3:**
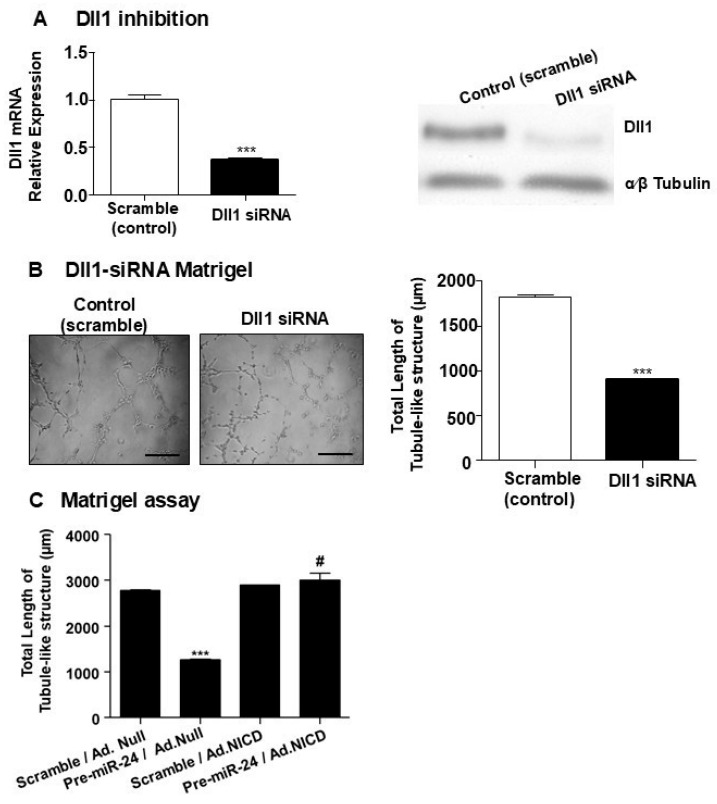
Dll1 inhibition in HUVECs affects the network-formation capability but NICD overexpression rescues it. Left bar graph (**A**, **left**) Dll1 mRNA relative expression in HUVECs transfected with Dll1-siRNA (black column) compared to HUVECs transfected with a scramble sequence (control). Dll1 mRNA expression was corrected to 18S and data were compared to the control group by the 2-ΔΔCt formula. Values are means ± SEM. *** *p* < 0.005 vs. scramble (**A**, **right**) Western blot bands of Dll1 protein and housekeeping α β-tubulin protein in HUVECs transfected with Dll1-siRNA compared to HUVECs transfected with a scramble sequence (control). (**B**, **left**) Photomicrographs of representative fields show the endothelial networks formed by HUVECs transfected with Dll1-siRNA compared to the control group (Scramble). (**B**, **right**) The bar graph represents the quantification of the total length of tube-like structures obtained in HUVECs transfected with Dll1-siRNA (black column) compared to the control group (scramble, white column). Values are means ± SEM. *** *p* < 0.005 vs. Scramble (**C**) The bar graph represents the quantification of the total length of tube-like structures in HUVECs treated as indicated: HUVECs transfected with scramble and infected with *Ad.Null*; transfected with pre-miR-24-3p and infected with *Ad.Null*; transfected with scramble and infected with *Ad.NICD* and HUVECs were transfected with pre-miR-24-3p and infected with *Ad.NICD*. Experiments were performed in triplicate and repeated 3 times. The raw data for the Panel (**C**) graph are presented in Supplemental Table S1. *** *p* < 0.005 vs. Scramble/*Ad.Null*; # *p* < 0.05 vs. miR-24 precursor/*Ad.Null*.

**Figure S2:**
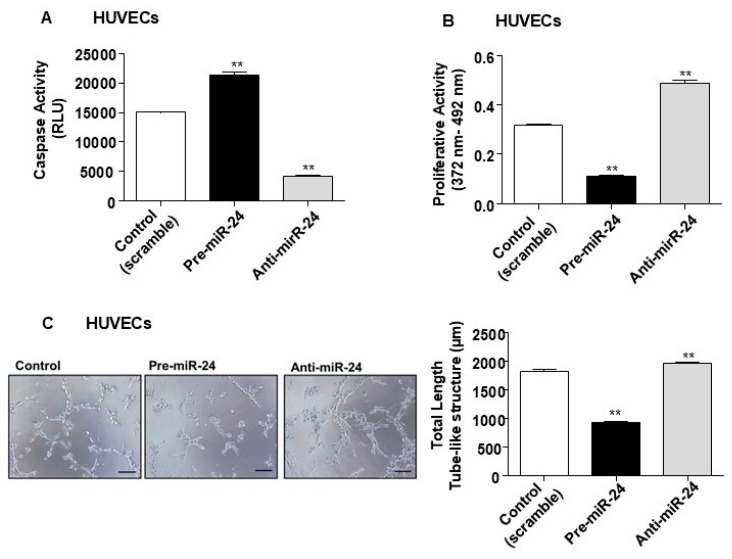
Apoptosis, Proliferation Assay, and networking on Matrigel Assay with overexpression and inhibition of miR-24 in HUVECs. The assays were performed after the transfection with miR-24 mimic (black bars) or miR-24 inhibitor (light grey bars), and a scramble was used as a control (white bars). Bar graph (**A**) shows apoptosis, evaluated by Caspase-GLO activity Assay, and bar graph. Experiments were performed in triplicate and repeated 3 times. Values are means ± SEM. ** *p* < 0.005 vs. Scramble. (**B**) shows the proliferative capability of HUVECs evaluated by BrdU incorporation. Photomicrographs of representative fields (**C**) show the endothelial network capability formation on Matrigel (scale bar 40 µm). RLU: Relative Luminescent Units. BrdU incorporation was evaluated analysing the absorbance between 370 and 492 nm. Experiments were performed in triplicate and repeated 3 times. Values are means ± SEM., ** *p* < 0.005 vs. Scramble.
