# Supplementary material for: MicroRNA-24-3p Targets Notch and Other Vascular Morphogens to Regulate Post-ischemic Microvascular Responses in Limb Muscles
Source: Int J Mol Sci. 2020 Mar 3;21(5):1733. doi: 10.3390/ijms21051733 (PMC7084374; doi:10.3390/ijms21051733)
Supplement: Supplementary file 1 [file ijms-21-01733-s001.pdf]

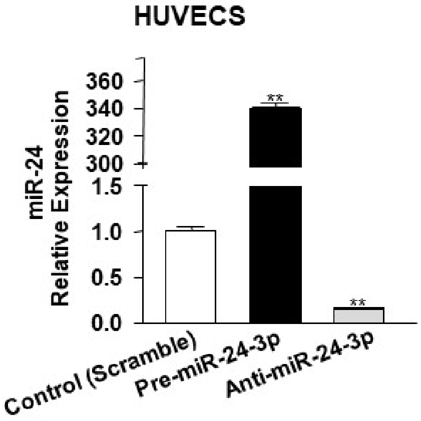

Supplementary Figure S1: In vitro miR-24-3p expressional modulation. miR-24-3p expression changes after 72 h post-transfection with either pre-miR-24-3p (miR-24-3p, black bar) or anti-miR-24-3p (miR-24-3plight grey column) compared to the control (scramble, white column). miR-24-3p expression was normalised to Snu6. Experiments were performed in triplicate and repeated three times. Values are means ± SEM. ** p < 0.01 vs. control.

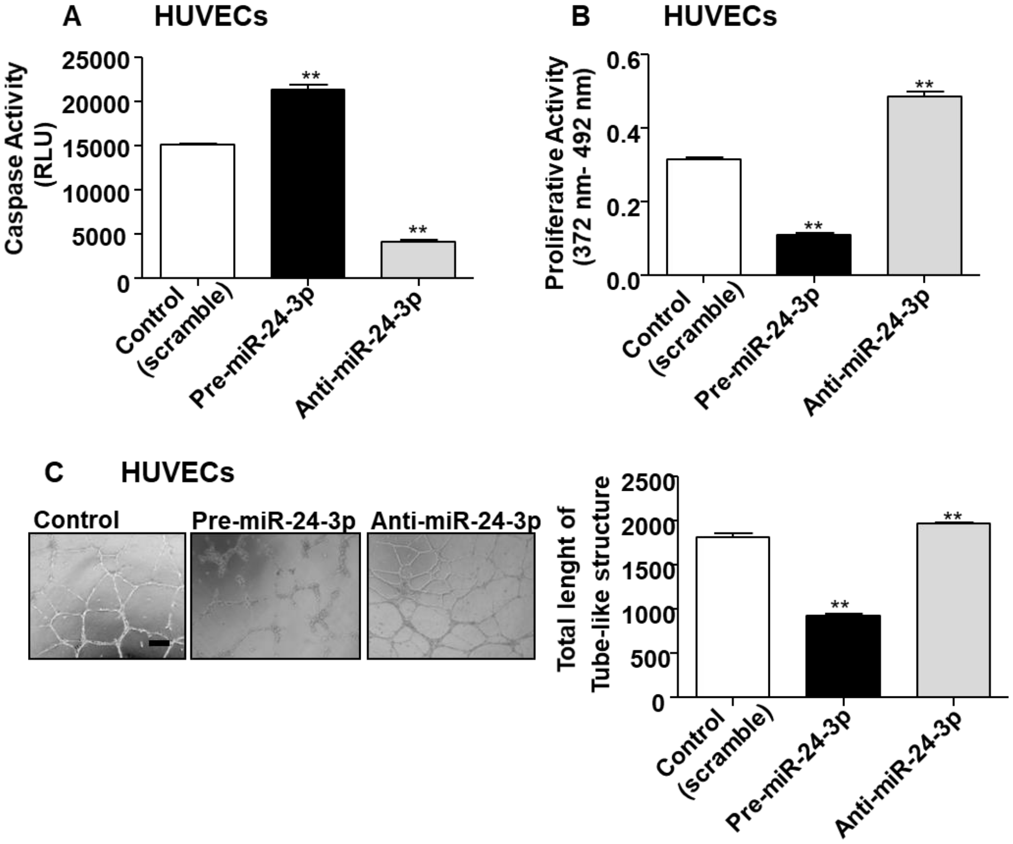

Supplementary Figure S2. Changes in the apoptosis, proliferation, and cord formation capacity induced by transfection of HUVECs with either pre-miR-24-3p (black bars) or anti-miR-24-3p (light grey bars) in comparison to a scramble control (white bars). (A) Cell apoptosis was evaluated by Caspase-GLO activity assay. RLU: relative luminescent units. (B) the proliferative capability of HUVECs evaluated by a BrdU incorporation assay (C) Photomicrographs of representative fields show the endothelial network capability formation on Matrigel (scale bar 40 μm). Experiments were performed in triplicate and repeated three times. Values are means ± SEM. **p < 0.01 vs. scramble.

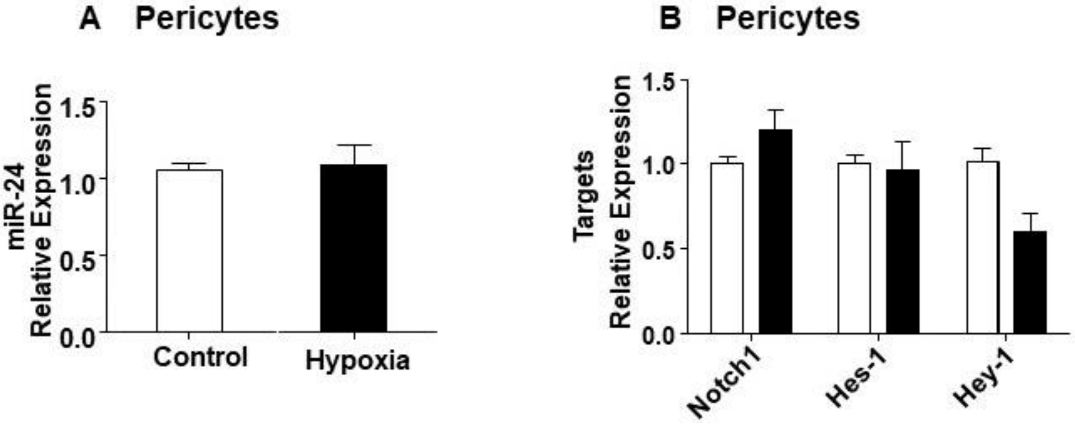

Supplementary Figure S3: Pericytes do not change miR-24-3p or Notch1 expression when exposed to hypoxia. Bar graph (**A**) shows miR-24-3p modulation at 48 h in hypoxia (black column) or normoxia (white column). Bar graph (**B**) shows Notch1 mRNA and Dll1 mRNA modulation at 48 h in hypoxia (black columns) or normoxia (white columns). miR-24-3p expression was corrected to Snu6, and data are compared to the matched time-point group by the 2ΔCt method. The mRNA expression of Notch1, Hes-1, and Hey-1 was corrected to 18S. Experiments were performed in triplicate and repeated three times. Values are means ± SEM.

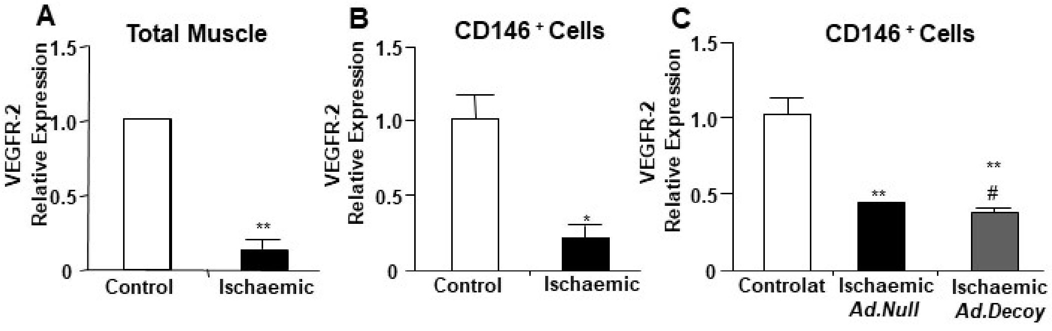

Supplementary Figure S4. VEGFR-2 relative expression at 3 days of L.I. in a murine model. VEGFR-2 relative expression at 3 days of L.I. VEGFR-2 relative expression in total ischemic muscle (black bar) compared to the control non-ischemic muscle (white bar) (A). Modulation of VEGFR-2 in ECs sorted from ischemic muscle (black bar) compared to the ECs sorted cells from control non-ischemic muscle (white bar) (B). Modulation on VEGF-R2 in ECs after *Ad. Decoy-miR-24-3p* treatment (grey bar) compared to *Ad. Null* treatment (black bar) and contralateral muscle (control, white bar) (C). VEGFR-2 expression, corrected to 18S, is significantly reduced compared to the control. Experiments were performed in triplicate and repeated five times. Values are means ± SEM. * p < 0,05; ** *p* < 0.01 vs. control.

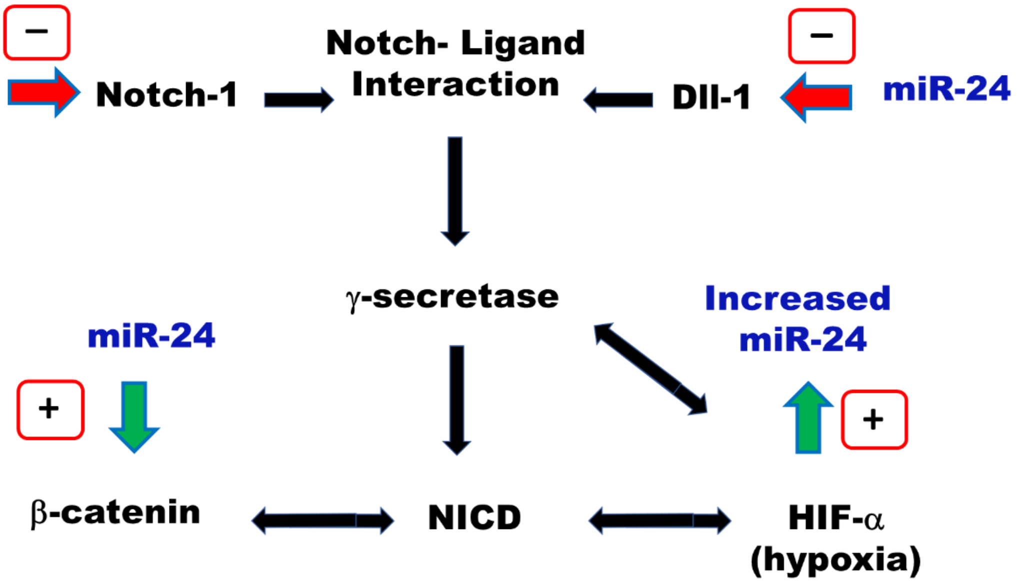

Supplementary Figure S5. Proposed model of the impact of miR-24-3p on the interaction between Notch and β-catenin in hypoxic endothelial cells. NICD interacts with β-catenin and HIF-1α. Moreover, HIF-1α increases the γ-secretase activity resulting in enhanced Notch signalling. miR-24-3p is upregulated under hypoxic conditions and can directly repress Notch-1 and Dll-1, while it can indirectly increase β-catenin. Green indicates expressional promotion and red expressional suppression. Double arrows indicate physical interaction.
